# A guide to selecting high-performing antibodies for ADNP (UniProt ID: Q9H2P0) for use in western blot, immunoprecipitation, and immunofluorescence

**DOI:** 10.12688/f1000research.160121.1

**Published:** 2024-12-20

**Authors:** Vera Ruíz Moleón, Charles Alende, Maryam Fotouhi, Riham Ayoubi, Sara González Bolívar, Carl Laflamme, NeuroSGC/YCharOS/EDDU collaborative group, ABIF consortium

**Affiliations:** 1 Department of Neurology and Neurosurgery, Montreal Neurological Institute-Hospital, Montreal, Québec, H3A 2B4, Canada

**Keywords:** Q9H2P0, ADNP, Activity-Dependent Neuroprotective Protein, Activity-Dependent Neuroprotector homeobox Protein, antibody characterization, antibody validation, western blot, immunoprecipitation, immunofluorescence

## Abstract

ADNP is a multifunctional protein involved in chromatin remodeling, transcription, and microtubule interaction, playing a critical role in brain development, with mutations linked to *ADNP*-Related Disorder. Here we have characterized seven ADNP commercial antibodies for western blot, immunoprecipitation, and immunofluorescence using a standardized experimental protocol based on comparing read-outs in knockout cell lines and isogenic parental controls. These studies are part of a larger, collaborative initiative seeking to address antibody reproducibility issues by characterizing commercially available antibodies for human proteins and publishing the results openly as a resource for the scientific community. While use of antibodies and protocols vary between laboratories, we encourage readers to use this report as a guide to select the most appropriate antibodies for their specific needs.

## Introduction

ADNP (Activity-Dependent Neuroprotector Homeobox Protein) is part of the SWI/SNF chromatin remodeling complex, a key regulator of RNA transcription and splicing. ^1^ It is a multifunctional protein characterized by nine Zinc fingers, a nuclear localization signal and a DNA-binding homeobox domain, ^2^ which collectively support its role as a transcription factor. ^3^ The protein also contains a small NAP motif (amino acids “NAPVSIPQ”) site that contributes to its interaction with microtubules. ^4^ Given its function in brain development ^5^ and other roles in the cell, ^6^ *ADNP* deletion is lethal in mice and haploinsufficiency results in cognitive impairment. ^7^ Mutations in the *ADNP* gene are associated with several congenital abnormalities and intellectual disabilities, including Helsmoortel-Van der Aa Syndrome ^8^ and *ADNP*-related Autism Spectrum Disorder, ^9^ collectively termed “ *ADNP*-Related Disorder”. ^10^

This research is part of a broader collaborative initiative in which academics, funders and commercial antibody manufacturers are working together to address antibody reproducibility issues by characterizing commercial antibodies for human proteins using standardized protocols, and openly sharing the data. ^11– 13^ Here we evaluated the performance of seven commercial antibodies for ADNP for use in western blot, immunoprecipitation, and immunofluorescence, enabling biochemical and cellular assessment of ADNP properties and function. The platform for antibody characterization used to carry out this study was endorsed by a committee of industry and academic representatives. It consists of identifying human cell lines with adequate target protein expression and the development/contribution of equivalent knockout (KO) cell lines, followed by antibody characterization procedures using most commercially available antibodies against the corresponding protein. The standardized consensus antibody characterization protocols are openly available on Protocol Exchange, a preprint server (DOI: 10.21203/rs.3.pex-2607/v1). ^14^

The authors do not engage in result analysis or offer explicit antibody recommendations. Our primary aim is to deliver top-tier data to the scientific community, grounded in Open Science principles. This empowers experts to interpret the characterization data independently, enabling them to make informed choices regarding the most suitable antibodies for their specific experimental needs. Guidelines on how to interpret antibody characterization data found in this study are featured on the YCharOS gateway. ^15^

## Results and discussion

Our standard protocol involves comparing readouts from wild type (WT) and KO cell lines. ^14, 16^ In the absence of commercially available KO cell lines, siRNA technology can be employed to knockdown (KD) the target gene. ^17, 18^ To determine which cell line demonstrates high expression of ADNP and thus be appropriate for KD, the first step is to identify a cell line that expresses sufficient levels of a given protein to generate a measurable signal using antibodies. To this end, we examined the DepMap (Cancer Dependency Map Portal, RRID:SCR_017655) transcriptomics database to identify cell lines that express the target at levels greater than 2.5 log _2_ (transcripts per million “TPM” + 1), which we have found to be a suitable cut-off. ^11^ As a result, DMS 53 which expresses the ADNP transcript at 5.7 was identified as a suitable cell line to use here. A non-targeting siRNA pool was used to treat the DMS 53 control (ctrl) cells, while *ADNP* was KD using a pool of siRNA targeting this gene.

**Figure 1.**
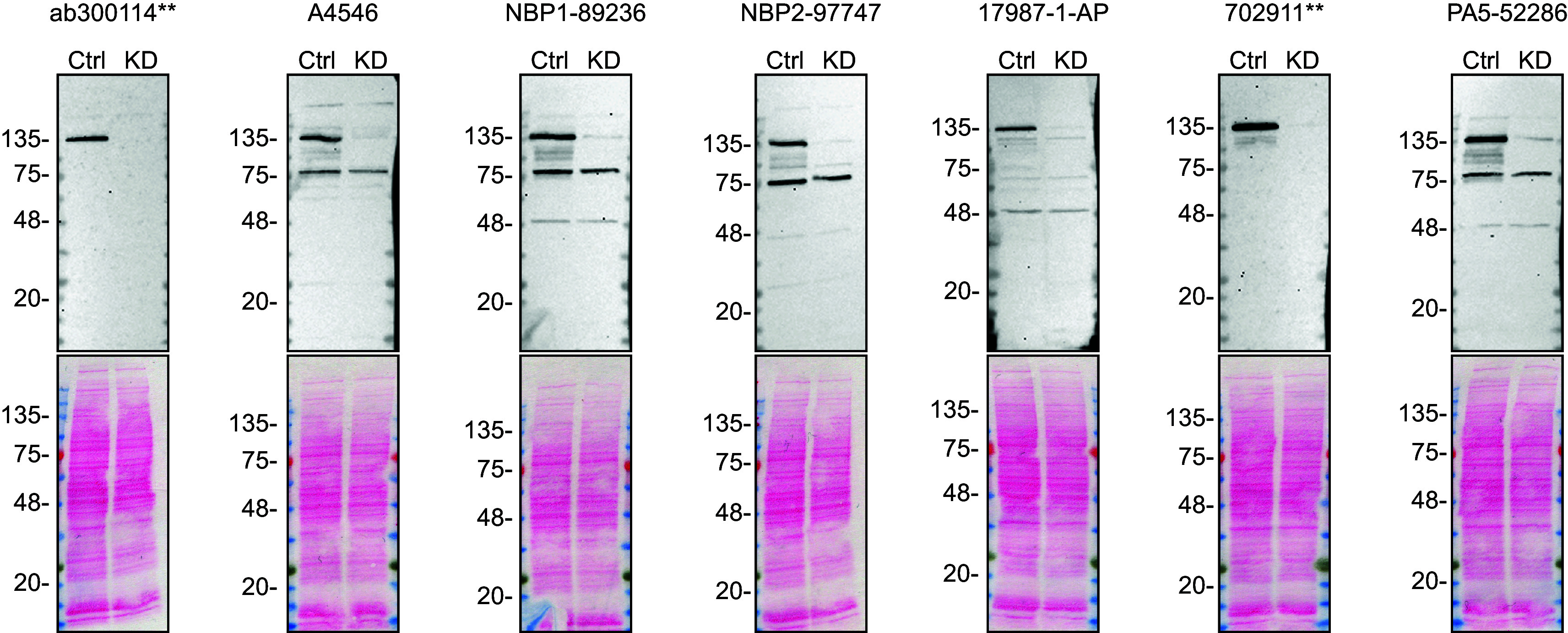
ADNP antibody screening by western blot. Lysates of DMS 53 ctrl and *ADNP* KD were prepared, and 30 μg of protein were processed for western blot with the indicated ADNP antibodies. The Ponceau stained transfers of each blot are presented to show equal loading of ctrl and KD lysates and protein transfer efficiency from the acrylamide gels to the nitrocellulose membrane. Antibody dilutions were chosen according to the recommendations of the antibody supplier. Antibody dilution used: ab300114** at 1/1000, A4546 at 1/1000, NBP1-89236 at 1/250 (0.4 μg/ml), NBP2-97747 at 1/1000, 17987-1-AP at 1/1000, 702911** at 1/1000, PA5-52286 at 1/250 (0.4 μg/ml). Predicted band size: predicted band size 123.5 kDa. **Recombinant antibody.

To screen all seven antibodies by western blot, ctrl and *ADNP* KD protein lysates were ran on SDS-PAGE, transferred onto nitrocellulose membranes, and then probed with the ADNP antibodies in parallel ( Figure 1).

**Figure 2.**
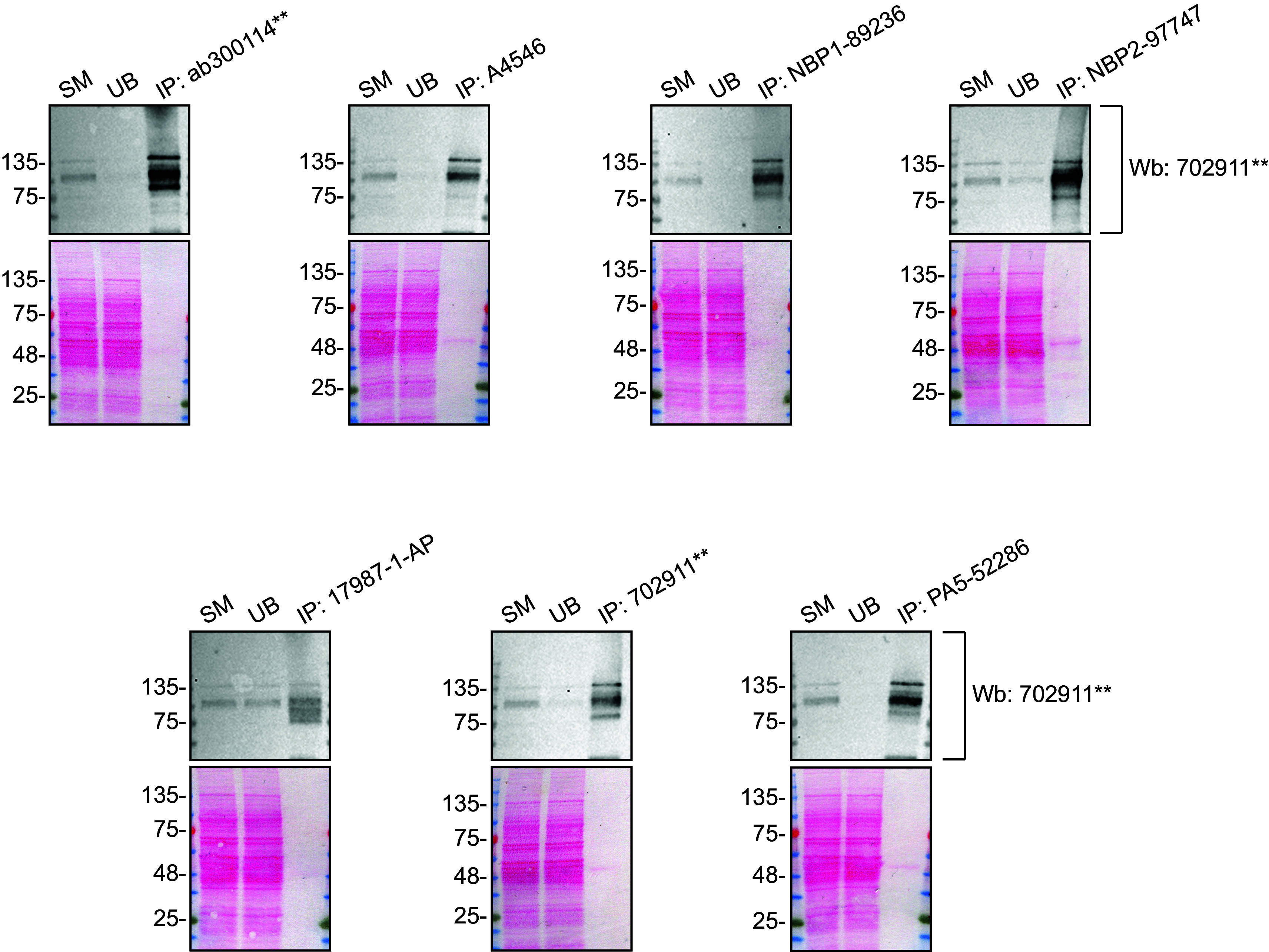
ADNP antibody screening by immunoprecipitation. DMS 53 WT lysates were prepared, and immunoprecipitation was performed using 1 mg of lysate and 2.0 μg of the indicated ADNP antibodies pre-coupled to Dynabeads protein A or protein G. Samples were washed and processed for western blot with the indicated ADNP antibody. For western blot, 702911** was used at 1/500. The Ponceau stained transfers of each blot are shown. SM=6% starting material; UB=6% unbound fraction; IP=immunoprecipitate, **Recombinant antibody.

We then assessed the capability of all seven antibodies to capture ADNP from DMS 53 protein extracts using immunoprecipitation techniques, followed by western blot analysis. For the immunoblot step, a specific ADNP antibody identified previously (refer to Figure 1) was selected. Equal amounts of the starting material (SM) and the unbound fractions (UB), as well as the whole immunoprecipitate (IP) eluates were separated by SDS-PAGE ( Figure 2).

**Figure 3.**
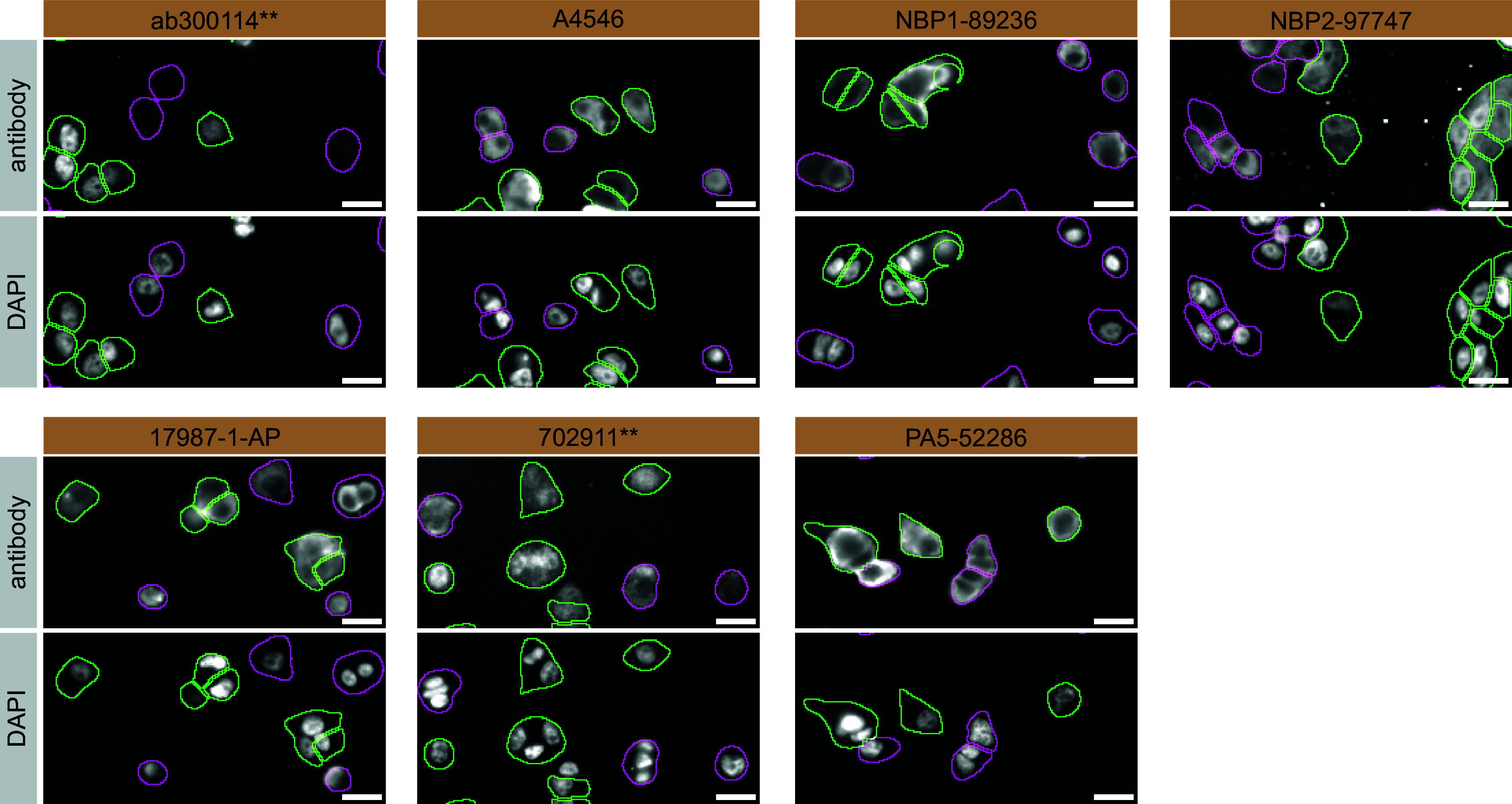
ADNP antibody screening by immunofluorescence. DMS 53 ctrl and *ADNP* KD cells were labelled with a green or a far-red fluorescent dye, respectively. Ctrl and KD cells were mixed and plated to a 1:1 ratio on coverslips. Cells were stained with the indicated ADNP antibodies and with the corresponding Alexa-fluor 555 coupled secondary antibody including DAPI. Acquisition of the blue (nucleus-DAPI), green (ctrl), red (antibody staining) and far-red (KD) channels was performed. Representative images of the merged blue and red (grayscale) channels are shown. Ctrl and KD cells are outlined with green and magenta dashed lines, respectively. When an antibody was recommended for immunofluorescence by the supplier, we tested it at the recommended dilution. The rest of the antibodies were tested at 1 and 2 μg/ml and the final concentration was selected based on the detection range of the microscope used and a quantitative analysis not shown here. Antibody dilution used: ab300114** at 1/500, A4546 at 1/700, NBP1-89236 at 1/100, NBP2-97747 at 1/300, 17987-1-AP at 1/600, 702911** at 1/500, PA5-52286 at 1/100. Bars = 10 μm. **Recombinant antibody.

For immunofluorescence, the seven antibodies were screened using a mosaic strategy. First, DMS 53 ctrl and *ADNP* KD cells were labelled with different fluorescent dyes in order to distinguish the two cell lines, and the ADNP antibodies were evaluated. Both ctrl and KD lines were imaged in the same field of view to reduce staining, imaging and image analysis bias ( Figure 3). Quantification of immunofluorescence intensity in hundreds of ctrl and KD cells was performed for each antibody tested, and the images presented in Figure 3 are representative of this analysis. ^14^

In conclusion, we have screened seven ADNP commercial antibodies by western blot, immunoprecipitation, and immunofluorescence by comparing the signal produced by the antibodies in human DMS 53 ctrl and *ADNP* KD cells. To assist users in interpreting antibody performanyce, Table 3 outlines various scenarios in which antibodies may perform in all three applications. ^11^ Several high-quality and renewable antibodies that successfully detect ADNP were identified in all applications. Researchers who wish to study ADNP in a different species are encouraged to select high-quality antibodies, based on the results of this study, and investigate the predicted species reactivity of the manufacturer before extending their research.

### Limitations

Inherent limitations are associated with the antibody characterization platform used in this study. Firstly, the YCharOS project focuses on renewable (recombinant and monoclonal) antibodies and does not test all commercially available ADNP antibodies. YCharOS partners provide approximately 80% of all renewable antibodies, but some top-cited polyclonal antibodies may not be available through these partners.

Secondly, the YCharOS effort employs a non-biased approach that is agnostic to the protein for which antibodies have been characterized. The aim is to provide objective data on antibody performance without preconceived notions about how antibodies should perform or the molecular weight that should be observed in western blot. As the authors are not experts in ADNP only a brief overview of the protein’s function and its relevance in disease is provided. ADNP experts are invited to analyze and interpret observed banding patterns in western blots and subcellular localization in immunofluorescence.

Thirdly, YCharOS experiments are not performed in replicates primarily due to the use of multiple antibodies targeting various epitopes. Once a specific antibody is identified, it validates the protein expression of the intended target in the selected cell line, confirms the lack of protein expression in the KO cell line and supports conclusions regarding the specificity of the other antibodies. All experiments are performed using master mixes, and meticulous attention is paid to sample preparation and experimental execution. In IF, the use of two different concentrations serves to evaluate antibody specificity and can aid in assessing assay reliability. In instances where antibodies yield no signal, a repeat experiment is conducted following titration. Additionally, our independent data is performed subsequently to the antibody manufacturers internal validation process, therefore making our characterization process a repeat.

Lastly, as comprehensive and standardized procedures are respected, any conclusions remain confined to the experimental conditions and cell line used for this study. The use of a single cell type for evaluating antibody performance poses as a limitation, as factors such as target protein abundance significantly impact results. ^14^ Additionally, the use of cancer cell lines containing gene mutations poses a potential challenge, as these mutations may be within the epitope coding sequence or other regions of the gene responsible for the intended target. Such alterations can impact the binding affinity of antibodies. This represents an inherent limitation of any approach that employs cancer cell lines.

## Method

The standardized protocols used to carry out this KO cell line-based antibody characterization platform was established and approved by a collaborative group of academics, industry researchers and antibody manufacturers. The detailed materials and step-by-step protocols used to characterize antibodies in western blot, immunoprecipitation and immunofluorescence are openly available on Protocol Exchange, a preprint server (DOI: 10.21203/rs.3.pex-2607/v1). ^14^ Brief descriptions of the experimental setup used to carry out this study can be found below.

### Cell lines and antibodies

Cell lines used and primary antibodies tested in this study are listed in Tables 1 and 2, respectively. To ensure that the cell lines and antibodies are cited properly and can be easily identified, we have included their corresponding Research Resource Identifiers, or RRID. ^19, 20^ DMS 53 cells were treated with the ON-TARGETplus Human ADNP siRNA from Horizon Discovery, cat. number L-012857-01-0005. Ctrl DMS 53 cells were treated with the ON-TARGETplus Non-targeting Control Pool, cat. number D-001810-10-05. Lipofectamine RNAiMAX (Thermo Fisher Scientific, cat. number 13778030) was used to transfect the siRNA following the manufacturer’s protocol.

**Table 1.** Summary of the cell lines used.

Institution	Catalog number	RRID (Cellosaurus)	Cell line	Genotype
ATCC	CRL-2062	CVCL_1177	DMS 53	WT

**Table 2.** Summary of the ADNP antibodies tested.

Company	Catalog number	Lot number	RRID (Antibody Registry)	Clonality	Clone ID	Host	Concentration (μg/μL)	Vendors recommended applications
Abcam	ab300114 **	1074869-4	AB_3097704	recombinant mono	EPR25434-4	rabbit	0.54	Wb, IP, IF
ABclonal	A4546	0092990201	AB_2765746	polyclonal	-	rabbit	0.70	Wb, IF
Bio-Techne (Novus Biologicals)	NBP1-89236	000036064	AB_11008573	polyclonal	-	rabbit	0.10	Wb, IF
Bio-Techne (Novus Biologicals)	NBP2-97747	HD12JL1810	AB_3094832	polyclonal	-	rabbit	n/a	Wb, IP, IF
Proteintech	17987-1-AP	00040236	AB_2222383	polyclonal	-	rabbit	0.60	Wb, IF
Thermo Fisher Scientific	702911 **	2485913	AB_2848220	recombinant mono	16H17L68	rabbit	0.50	Wb, IF
Thermo Fisher Scientific	PA5-52286	YE3914208	AB_2637725	polyclonal	-	rabbit	0.10	Wb, IF

Wb = western blot; IP = immunoprecipitation; IF = immunofluorescence.

** Recombinant antibody, n/a=not available.

**Table 3.** Illustrations to assess antibody performance in all western blot, immunoprecipitation and immunofluorescence.

Western blot	Immunoprecipitation	Immunofluorescence
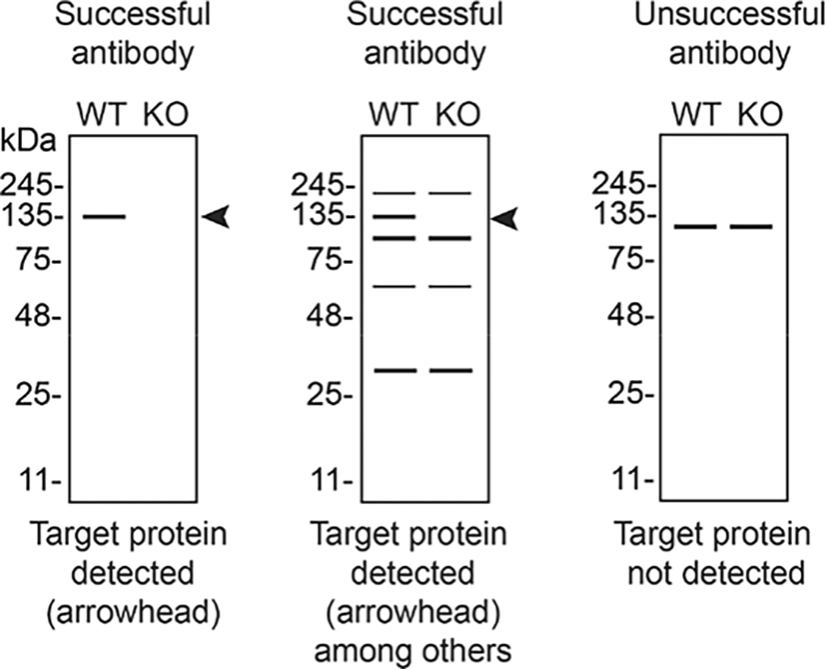	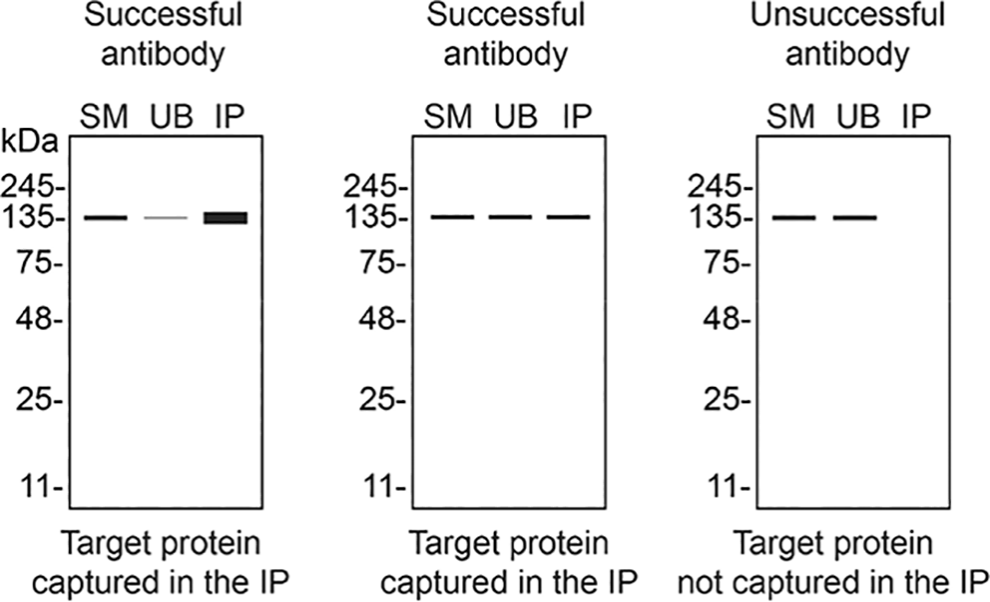	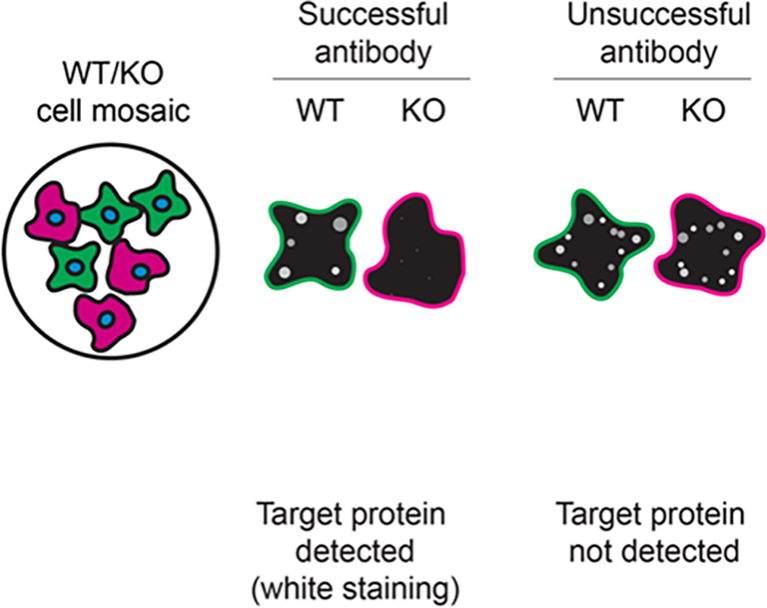

This table has been reproduced with permission from Ayoubi et al., Elife, 2023. ^11^

Peroxidase-conjugated goat anti-rabbit antibody is from Thermo Fisher Scientific, cat. number 65-6120. Alexa-555-conjugated goat anti-rabbit secondary antibody is from Thermo Fisher Scientific, cat. number A-21429. Peroxidase-conjugated Protein A for IP detection is from Cell Signaling Technology, cat. number 12291.

### Antibody screening by western blot

DMS 53 ctrl and *ADNP* KD cells were collected in RIPA buffer (25mM Tris-HCl pH 7.6, 150 mM NaCl, 1% NP-40, 1% sodium deoxycholate, 0.1% SDS) (Thermo Fisher Scientific, cat. number 89901) supplemented with 1× protease inhibitor cocktail mix (MilliporeSigma, cat. number P8340). Lysates were sonicated briefly and incubated 30 min on ice. Lysates were spun at ~110,000 × *g* for 15 min at 4°C and equal protein aliquots of the supernatants were analyzed by SDS-PAGE and western blot. BLUelf prestained protein ladder (GeneDireX, cat. number PM008-0500) was used.

Western blots were performed with precast midi 4-20% Tris-Glycine polyacrylamide gels (Thermo Fisher Scientific, cat. number WXP42012BOX) ran with Tris/Glycine/SDS buffer (Bio-Rad, cat. number 1610772), loaded in Laemmli loading sample buffer (Thermo Fisher Scientific, cat. number AAJ61337AD) and transferred on nitrocellulose membranes. Proteins on the blots were visualized with Ponceau S staining (Thermo Fisher Scientific, cat. number BP103-10) which is scanned to show together with individual western blot. Blots were blocked with 5% milk for 1 hr, and antibodies were incubated O/N at 4°C with 5% milk in TBS with 0,1% Tween 20 (TBST) (Cell Signalling Technology, cat. number 9997). Following three washes with TBST, the peroxidase conjugated secondary antibody was incubated at a dilution of ~0.2 μg/ml in TBST with 5% milk for 1 hr at room temperature followed by three washes with TBST. Membranes were incubated with Pierce ECL (Thermo Fisher Scientific, cat. number 32106) or Clarity Western ECL Substrate (Bio-Rad, cat. number 1705061) prior to detection with the iBright™ CL1500 Imaging System (Thermo Fisher Scientific, cat. number A44240).

### Antibody screening by immunoprecipitation

Antibody-bead conjugates were prepared by adding 2.0 μg to 500 μl of Pierce IP Lysis Buffer from Thermo Fisher Scientific (cat. number 87788) in a microcentrifuge tube, together with 30 μl of Dynabeads protein A- (rabbit antibodies) (Thermo Fisher Scientific, cat. number 10002D). Antibody NBP2-97747 is at an unknown concentration, and 20 μl were tested in the IP. Tubes were rocked for ~1 h at 4°C followed by two washes to remove unbound antibodies.

DMS 53 WT were collected in Pierce IP buffer (25 mM Tris-HCl pH 7.4, 150 mM NaCl, 1 mM EDTA, 1% NP-40 and 5% glycerol) supplemented with protease inhibitor. Lysates were rocked 30 min at 4°C and spun at 110,000 × *g* for 15 min at 4°C. 0.5 ml aliquots at 2 mg/ml of lysate were incubated with an antibody-bead conjugate for ~1 h at 4°C. The unbound fractions were collected, and beads were subsequently washed three times with 1.0 ml of IP buffer and processed for SDS-PAGE and western blot on precast midi 4-20% Tris-Glycine polyacrylamide gels. Protein A:HRP was used as a secondary detection system at a concentration of 0.5 μg/ml.

### Antibody screening by immunofluorescence

DMS 53 ctrl and *ADNP* KD cells were labelled with a green and a far-red fluorescence dye, respectively (Thermo Fisher Scientific, cat. number C2925 and C34565). The nuclei were labelled with DAPI (Thermo Fisher Scientific, cat. Number D3571) fluorescent stain. Ctrl and KD cells were plated on 96-well plate with optically clear flat-bottom (Perkin Elmer, cat. number 6055300) as a mosaic and incubated for 24 hrs in a cell culture incubator at 37 ^o^C, 5% CO _2_. Cells were fixed in 4% paraformaldehyde (PFA) (VWR, cat. number 100503-917) in phosphate buffered saline (PBS) (Wisent, cat. number 311-010-CL). Cells were permeabilized in PBS with 0,1% Triton X-100 (Thermo Fisher Scientific, cat. number BP151-500) for 10 min at room temperature and blocked with PBS with 5% BSA, 5% goat serum (Gibco, cat. number 16210-064) and 0.01% Triton X-100 for 30 min at room temperature. Cells were incubated with IF buffer (PBS, 5% BSA, 0,01% Triton X-100) containing the primary ADNP antibodies overnight at 4°C. Cells were then washed 3 × 10 min with IF buffer and incubated with corresponding Alexa Fluor 555-conjugated secondary antibodies in IF buffer at a dilution of 1.0 μg/ml for 1 hr at room temperature with DAPI. Cells were washed 3 × 10 min with IF buffer and once with PBS.

Images were acquired on an ImageXpress micro confocal high-content microscopy system (Molecular Devices), using a 20× NA 0.95 water immersion objective and scientific CMOS cameras, equipped with 395, 475, 555 and 635 nm solid state LED lights (lumencor Aura III light engine) and bandpass filters to excite DAPI, Cellmask Green, Alexa-555 and Cellmask Red, respectively. Images had pixel sizes of 0.68 × 0.68 microns, and a z-interval of 4 microns. For analysis and visualization, shading correction (shade only) was carried out for all images. Then, maximum intensity projections were generated using 3 z-slices. Segmentation was carried out separately on maximum intensity projections of Cellmask channels using CellPose 1.0, and masks were used to generate outlines and for intensity quantification. ^21^ Figures were assembled with Adobe Illustrator.

## Data Availability

Zenodo: Antibody Characterization Report for ADNP, https://doi.org/10.5281/zenodo.14366335. ^22^ Zenodo: Dataset for the ADNP antibody screening study, https://doi.org/10.5281/zenodo.14423002. ^23^ Data are available under the terms of the Creative Commons Attribution 4.0 International license (CC-BY 4.0).
