# Supplementary material for: A guide to selecting high-performing antibodies for ADNP (UniProt ID: Q9H2P0) for use in western blot, immunoprecipitation, and immunofluorescence
Source: F1000Res. 2024 Dec 20;13:1545. [Version 1] doi: 10.12688/f1000research.160121.1 (PMC11822250; doi:10.12688/f1000research.160121.1)
Supplement: Peer review history

# Reviewer report: Reviewer response for version 1

**DOI:** 10.5256/f1000research.175953.r352668

**Published:** 2025-02-12

**Authors:** Illana Gozes, Maram Ganaiem, Artur Galushkin

**Affiliations:** 1 Tel Aviv University, Tel Aviv, Israel; 2 Faculty of Medical and Health Sciences, Tel Aviv University (Ringgold ID: 26745), Tel Aviv, Israel

**Review of “A Guide to Selecting High-Performing Antibodies for ADNP”**

**Overview**

 The article under review provides a characterization of seven commercially available antibodies targeting ADNP. The study aims to address the widespread issue of antibody reproducibility by evaluating antibody performance on western blots, immunoprecipitations, and immunofluorescence. This open-access initiative seeks to guide researchers in selecting reliable antibodies for their specific experimental applications.

 Previously, we produced our own antibodies as well as used different commercial ADNP antibodies including for example the F-9 mouse monoclonal IgG1s (sc-376674, Santa Cruz) recognizing amino acids 1-138, mapped to the N-terminus of human ADNP (please see selected citations). These antibodies were not used for comparison here.

 The antibodies used in the current paper bring forth two recombinant monoclonal ADNP antibodies (rabbit), which seem to be of further interest in terms of specificity in western blots. Antibody 702911** (commercially available here - Ref:6 - was used at 1/500 for further validation of the immunoprecipitation results. The picture for the other recombinant antibody used (ab300114) looks very similar to the picture depicted on the commercial site: https://www.abcam.com/en-us/products/primary-antibodies/adnp-antibody-epr25434-4-ab300114?srsltid=AfmBOorKkVs4moqf5KOeCqUNZLbuTpM7BipS36RTsmMo9bpSixSbPt8k

 Interestingly, the ADNP molecular weight using the immunoprecipitation buffer (25 mM Tris-HCl pH 7.4, 150 mM NaCl, 1 mM EDTA, 1% NP-40 and 5% glycerol) seems lower than that seen for in the cell lysates utilizing the RIPA buffer (25mM Tris-HCl pH 7.6, 150mM NaCl, 1% NP-40, 1% sodium deoxycholate, 0.1% SDS), suggesting instability of the ADNP protein, although in both cases supplementation with protease inhibitors was mentioned – i.e. 1x protease inhibitor cocktail mix (MilliporeSigma, cat. number P8340). In this respect, the stability of the ADNP protein was recently addressed (please see selected citations).

**Key Findings and Strengths**

 The authors systematically evaluated ADNP antibodies using a standardized protocol in a DMS 53 cell line (DMS 53 is a **cell line exhibiting epithelial morphology** that was isolated in 1974 from the lung of a 54-year-old, White male patient with carcinoma. This cell line was deposited by OS Pettengill, G Sorenson, and can be used in cancer research). To address specificity ADNP expression was knocked down using siRNA. As mentioned above, performance was assessed across three methodologies:

- **Western blot:** Antibodies were screened for specificity based on expected molecular weights and signal intensity. Interestingly, the authors consistently detected a 135kD ADNP-immunoreactive band
- **Immunoprecipitation:** The ability of antibodies to pull down ADNP from cell lysates was examined.
- **Immunofluorescence:** Quantitative imaging revealed differential staining in control versus knockdown cells.

The study's strengths lie in its rigorous methodology, adherence to reproducibility standards, and commitment to Open Science. The authors provide openly available protocols and raw data, encouraging independent validation and application in diverse experimental contexts.

**Limitations and Gaps**

 The authors do not perform replicates, citing the use of multiple antibodies targeting various epitopes as the reason for this choice. They explain that validation of a single antibody provides sufficient confirmation of target specificity in the selected cell line and knockdown conditions. While this approach streamlines the process, it could reduce confidence in reproducibility, particularly for users requiring robust replication. The molecular weight obtained depends on post-translational modifications as well as protein sources, which could be further discussed.

**Methodological Assessment**

 While the use of a single cell line (DMS 53) ensures a controlled comparison, it introduces potential limitations in generalizability.

**Antibody Validation**

 The characterization revealed both high- and low-performing antibodies, allowing researchers to make informed choices. However, the article refrains from recommending specific antibodies, placing the onus on users to interpret the data. This non-prescriptive approach aligns with Open Science principles but might challenge researchers unfamiliar with antibody validation intricacies.

**Suggestions for Improvement**

 To increase the impact of future studies, the authors could:

- Include additional cell lines and tissue samples to validate findings across biological contexts (e.g., brain tissue).
- Conduct replicative studies to reinforce confidence in antibody reproducibility.
- Provide more detailed guidance on interpreting banding patterns and staining artifacts in western blot and immunofluorescence.

**Conclusion**

 This study offers a valuable resource for researchers investigating ADNP and related pathways. Despite some methodological constraints, it sets a high standard for antibody characterization. Incorporating these findings into experiments could streamline the selection of reliable tools and enhance the reproducibility of research.

 The recombinant antibodies show the highest specificity to ADNP and look promising to use.

 In writing this review I (Professor Illana Gozes) consulted with my two excellent PhD students as follows. Artur Galushkin is supported by a Marie Skłodowska-Curie TClock4AD fellowship aimed to discover pharmacological drugs that improve Alzheimer’s disease disrupted circadian rhythmicity & student training (double doctorate degrees). Project#: 101072895, EUROPEAN RESEARCH EXECUTIVE AGENCY (REA) REA) and Maram Ganaiem is supported by a Neubauer Family Foundation Student Scholarship at Tel Aviv University.

Are sufficient details of methods and materials provided to allow replication by others?

Yes

Is the rationale for creating the dataset(s) clearly described?

Yes

Are the datasets clearly presented in a useable and accessible format?

Yes

Are the protocols appropriate and is the work technically sound?

Yes

Reviewer Expertise:

Molecular and translational neuroscience/neurochemistry. Expert (discovorer) of ADNP.

We confirm that we have read this submission and believe that we have an appropriate level of expertise to confirm that it is of an acceptable scientific standard.

## References

[1] : Distinct Impairments Characterizing Different ADNP Mutants Reveal Aberrant Cytoplasmic-Nuclear Crosstalk. *Cells* .2022;11(19) : 10.3390/cells11192994 10.3390/cells11192994 36230962 PMC9563912

[2] : Introducing ADNP and SIRT1 as new partners regulating microtubules and histone methylation. *Mol Psychiatry* .2021;26(11) : 10.1038/s41380-021-01143-9 6550-6561 10.1038/s41380-021-01143-9 33967268

[3] : SH3- and actin-binding domains connect ADNP and SHANK3, revealing a fundamental shared mechanism underlying autism. *Mol Psychiatry* .2022;27(8) : 10.1038/s41380-022-01603-w 3316-3327 10.1038/s41380-022-01603-w 35538192

[4] : Activity-dependent neuroprotective protein (ADNP) differentially interacts with chromatin to regulate genes essential for embryogenesis. *Dev Biol* .2007;303(2) : 10.1016/j.ydbio.2006.11.039 814-24 10.1016/j.ydbio.2006.11.039 17222401

[5] : Tracing the invisible mutant ADNP protein in Helsmoortel-Van der Aa syndrome patients. *Sci Rep* .2024;14(1) : 10.1038/s41598-024-65608-x 14710 10.1038/s41598-024-65608-x 38926592 PMC11208605

[6] : Toxicity of pyrolysis products of thermal-resistant plastics including polyamide and polyester. *Nihon Eiseigaku Zasshi* .1978;33(2) : 10.1265/jjh.33.450 450-8 10.1265/jjh.33.450 702911

# Reviewer report: Reviewer response for version 1

**DOI:** 10.5256/f1000research.175953.r352667

**Published:** 2025-01-15

**Authors:** Christian Peter Moritz

**Affiliations:** 1 Department of Neurology, University hospital of Saint-Etienne, Saint-Etienne, France

This report evaluates a study focused on characterizing commercial ADNP antibodies across various applications, contributing to a broader initiative aimed at addressing antibody reproducibility issues.

 The project is highly relevant, addressing a critical issue in biomedical research by tackling the reproducibility crisis in antibody-based experiments. The use of standardized protocols in knockout and isogenic parental controls highlights a rigorous and transparent approach, offering valuable insights and an open-access resource for the scientific community.

 I see options for the following improvements:

 Major Points:

1. Figure 2: If I am not mistaken, the band pattern in all of the panels’ SM (start material) lanes should match the pattern observed in the control lane for antibody 702911 in Figure 1—specifically, a strong band around 135 kDa and a very weak band slightly below it. However, the pattern and the molecular weight of the main band differ, suggesting significant technical variations among the experiments. Can we rule out technical variability among the tested antibodies? Are the observed differences truly attributable to the antibodies themselves?
2. Precipitation Studies: For the precipitation studies, the authors used antibody 702911 to detect the precipitated proteins (“a specific ADNP antibody identified previously (refer to Figure 1) was selected”).
  However, in my view, antibody ab300114 appears to be more specific in Figure 1. I would like to understand why the authors chose antibody 702911 over ab300114
3. Competing Interests: Regarding their general YCharOS project, the authors might critically consider whether there are potential flaws in the study design due to the fact that the industry partner provided both the antibodies and the corresponding KO cell line. This creates a situation where the commercial antibody is being tested using a tool supplied by the same company. Are there any potential issues with this approach that should be discussed?
  I am aware that this criticism is rather for the general project as specifically for this study as the authors prepared their own Knock-down instead of using a company's Knock-out line if I understand it correctly. 

Minor Points:

1. Title and Abstract: I suggest introducing abbreviations—even for protein names like ADNP—the first time they are mentioned.
2. While many reviewers recommend using housekeeping proteins as loading controls for Western blots, I appreciate that the authors have opted for a total protein staining technique (Ponceau Red) to provide a more transparent representation of the loaded protein amounts (cf. 10.1002/pmic.201600189). This is just a positive note; no changes are necessary.

My best wishes to the authors for their valuable project.

Are sufficient details of methods and materials provided to allow replication by others?

Yes

Is the rationale for creating the dataset(s) clearly described?

Yes

Are the datasets clearly presented in a useable and accessible format?

Yes

Are the protocols appropriate and is the work technically sound?

Partly

Reviewer Expertise:

Antibodies, western blot, immunoprecipitation, immunofluorescence, ELISA, Proteomics, Neuropathies

I confirm that I have read this submission and believe that I have an appropriate level of expertise to confirm that it is of an acceptable scientific standard, however I have significant reservations, as outlined above.

# Reviewer report: Reviewer response for version 1

**DOI:** 10.5256/f1000research.175953.r352666

**Published:** 2025-01-10

**Authors:** Richard O'Kennedy

**Affiliations:** 1 Dublin City University, Dublin, Ireland

1. This paper presents data on the binding characteristics of a set of commercially available antibodies that bind to the protein ADNP (UniProt ID:Q9H2PO), as determined using a variety of techniques including western blotting, immunoprecipitation, and immunofluorescence employing standardized experimental devised protocols. Determinations were made  by comparing read-outs in knockout cell lines and isogenic parental controls.

 2. The paper is designed to present the resultant data to enable researchers to compare the  characteristics of the antibodies and their performance, in order to help researchers choose the most ideal/appropriate antibody for their particular applications.

 3. For the most part the data is clearly set out but it would have helped if the legends to the figures were more explanatory and included the full-names associated with abbreviations.

 4. The authors cite the approaches used in the methodologies employed. In addition, a section of the paper critically analyses the approaches and sets out possible shortcomings that the approaches may inherently have. This is very useful and would be particularly valuable if more papers had such evaluations generally.

 5. Ensuring that the binding characteristics/specificity of antibodies is as well defined as possible is very important and this paper certainly assists in this in relation to the target protein. Fundamental questions have been raised about data generated where the antibodies were poorly characterized, and had specificity issues that were later realized, which may have been avoided by more intense and careful binding characterization studies.

Are sufficient details of methods and materials provided to allow replication by others?

Yes

Is the rationale for creating the dataset(s) clearly described?

Yes

Are the datasets clearly presented in a useable and accessible format?

Yes

Are the protocols appropriate and is the work technically sound?

Yes

Reviewer Expertise:

Antibody production, characterisation and use with particular emphasis on diagnostics.

I confirm that I have read this submission and believe that I have an appropriate level of expertise to confirm that it is of an acceptable scientific standard.
